# Scattering Forces within a Left-Handed Photonic Crystal

**DOI:** 10.1038/srep41014

**Published:** 2017-01-23

**Authors:** Angeleene S. Ang, Sergey V. Sukhov, Aristide Dogariu, Alexander S. Shalin

**Affiliations:** 1 The International Research Centre for Nanophotonics and Metamaterials, ITMO University, St Petersburg, Russia; 2 CREOL, The College of Optics and Photonics, University of Central Florida, Orlando, Florida, USA; 3 Kotel’nikov Institute of Radio Engineering and Electronics of Russian Academy of Sciences (Ulyanovsk branch), Ulyanovsk, Russia; 4 Ulyanovsk State University, Ulyanovsk, Russia

## Abstract

Electromagnetic waves are known to exert optical forces on particles through radiation pressure. It was hypothesized previously that electromagnetic waves inside left-handed metamaterials produce negative radiation pressure. Here we numerically examine optical forces inside left-handed photonic crystals demonstrating negative refraction and reversed phase propagation. We demonstrate that even though the direction of force might not follow the flow of energy, the positive radiation pressure is maintained inside photonic crystals.

In his seminal paper^1^, Veselago described the properties of media with negative refractive index that later received the name “left-handed metamaterials” (LHM). The unique property of LHM is to bend the refracted rays in unusual way (negative refraction), the property that can be used to create plane-parallel lenses and to achieve super-resolved imaging. Besides many other unusual properties (subdiffraction focusing, reversed Cherenkov radiation, inversed Doppler effect, reversed Goos–Hanchen shift etc.), it was also suggested that LHM possess the property of counter propagating energy flux (Poynting vector **S**) and electromagnetic momentum **p** (let us note that the concept of back propagating waves was suggested even before Veselago^2^). According to Veselago, the reverse propagation of **p** would create negative (against the propagation of energy) forces acting on an object placed inside the left-handed metamaterial. Since its initial publication, this statement was discussed in many papers and the general conclusion among the scientific community is that these negative forces should exist^3^,^4^,^5^. Indeed, the scattering force acting on an object is defined by a change in a photon momentum during scattering, thus rendering the Veselago’s proposition as quite probable (see Supplementary Materials for example of optical force calculation inside left-handed metamaterial). And still, the discussion about the existence of such negative forces continues^3^,^4^,^5^,^6^. First designs for left-handed metamaterials based on split-ring resonators were suggested in 2000 by Pendry^7^. Since then, many designs have been developed and implemented^8^. However, the question about the existence of negative forces inside LHM remains unsettled in part, because the direct experimental verification of negative forces inside LHM is still impossible as all the proposed metamaterials are solid structures that prevent any movement for probe particles inside.

Soon after first metamaterial designs were proposed, left handed behavior and negative refraction were suggested for photonic crystals (PhC). Photonic crystals are periodic structures that create the same environment for photons as usual crystals do for electrons. In particular, bandgaps can exist in PhC for certain frequencies preventing photon propagation inside. For PhCs, it was also shown that left-handed negative refraction is possible in higher bands around the Γ point^9^,^10^,^11^,^12^. Under these conditions, **S** and **p** vectors can be antiparallel like in Veselago’s metamaterials. The crucial difference between PhCs and metamaterials described using an effective medium approach^11^,^13^ is that PhCs consist of elements larger or comparable in size to a wavelength. The gaps or empty spaces between those elements are also relatively large such that, in principle, one could place probe particles inside for the direct testing of mechanical action of light and addressing the important question of how optical forces behave in negative-refractive medium. In present paper we develop this concept with the aim of testing the presence of negative forces inside left-handed PhCs.

The presence of negative optical forces inside or in a vicinity of certain photonic structures was discussed previously in a number of papers. For example, negative forces were proposed to exist at the edge of a PhC waveguides^8^ or on a surface of regular waveguides^14^ due to surface waves and mode transformations. The use of surface plasmon-polaritons to achieve negative (pulling) force was suggested in refs 13 and 15. Optical pulling forces were also observed in homogeneous hyperbolic metamaterials due to excitation of extraordinary modes^13^ and in nanorod metamaterials with finite unite cell^16^. Negative forces due to backward waves were suggested for waveguides with high dielectric contrast^17^,^18^ or for birefringent materials^17^,^18^. Besides, enhanced optical forces due to slow light inside PhC waveguide were demonstrated^19^. The existence of negative optical force was found in certain three-dimensional photonic crystals^5^. However, it is not clear from that investigation whether the force is determined by the momentum exchange (nonconservative force) or by the gradient of electric field (local conservative force). Thus, till now the behavior of nonconservative optical forces in PhC demonstrating negative refractive index remained unexplored. We address this important question in present theoretical study.

## Probing nonconservative optical forces with a dipolar particle

**Figure 1:**
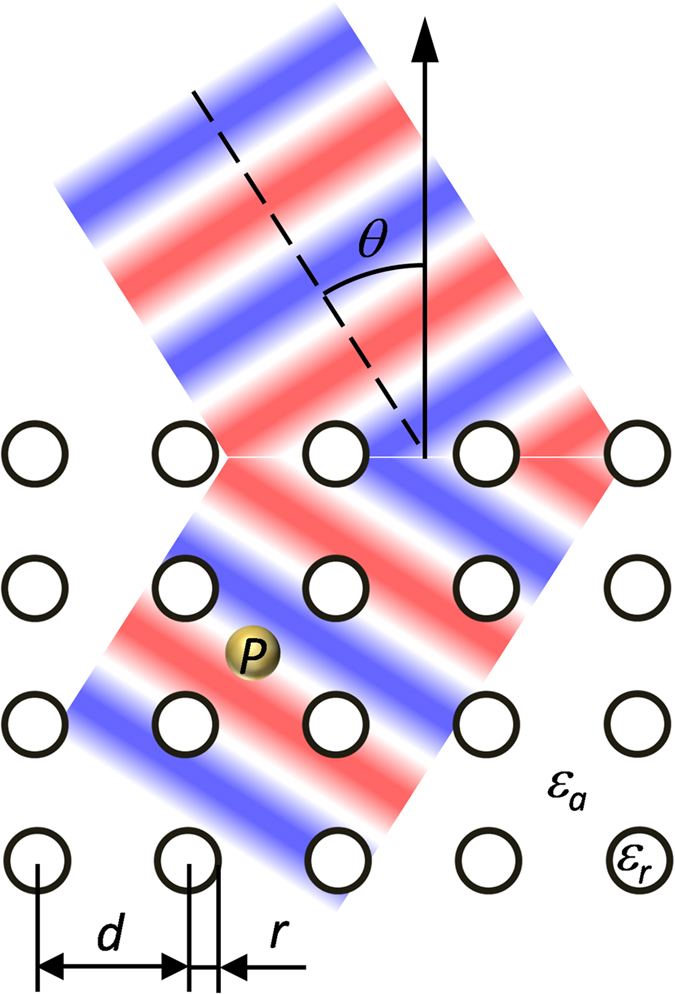
The geometry of the problem. External wave is incident at angle *θ* onto the surface of left-handed photonic crystal. Dipole particle *P* serves as a probe for local optical forces.

Using existing materials, such as periodically-arranged Al or GaAs rods in air, it is possible to fabricate PhC in which phase of the field propagates in opposite direction to the energy flow. The example of such negative phase propagation can be seen in Supplementary Media File 1 for the parameters of PhC taken from ref. 20. For PhC slabs, this usually (but, not always^20^) corresponds to the refraction of incident electromagnetic field in negative direction (Fig. 1). To date, several designs of PhC with negative refraction were proposed based on two-dimensional square^20^,^21^,^22^,^23^ and triangular^10^,^24^ lattices of circular rods. We summarized the proposed designs in Table 1. The field inside the PhC is characterized by isofrequency analysis. The band structure and isofrequency contours in the reviewed works are usually found using plane wave expansion method^25^ and negative refraction is confirmed by finite-difference time domain methods. The experimental demonstration of negative refraction is also presented in several papers^22^,^23^,^24^.

We consider semi-infinite two-dimensional photonic crystal structure consisting of rods with dielectric permittivity *ε*_*r*_ arranged in one of the lattices described in Table 1 (Fig. 1). The dielectric permittivity of the host material is *ε*_*a*_. External plane wave of frequency *f* is incident onto the interface of PhC structure under conditions providing left-handed negative refraction. To test the forces in PhC, we place inside a small dipole-like particle with dimensions allowing its free motion through the structure (Fig. 1). The time-averaged total force acting on a dipole is^26^,^27^


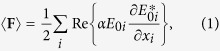


where *α* = *α′* + *iα″* is the complex particle’s polarizability, **E**_0_ is the field in the location of the particle. Disregarding changes in the field’s polarization, Eq. (1) can be rewritten as following^28^


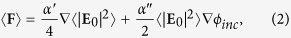


where ***ϕ***_*inc*_ is the phase of the field **E**_0_. The first term in Eq. (2) corresponds to the conservative gradient force. The second term corresponds to the nonconservative force, which can be regarded as a consequence of momentum transfer from the field to the particle. The latter force can cause unrestricted particle’s motion and this is the part of the force we are interested in. Negative phase velocity inside PhC^10^,^20^,^21^,^23^,^24^ corresponds to the negative gradient of phase in Eq. (2) and thus can potentially lead to negative forces in some cases.

Left-handed PhC suggested in a literature are composed of materials with high dielectric permittivity. Large variations of dielectric permittivity in PhC result in strong field inhomogeneities. This may cause the probe particles to be drawn to the locations of high field intensity due to the large first term in the right hand side of Eq. (2). However, one can see from Eq. (2) that different summands could be dominant for particles with different material properties (different polarizabilities)^29^,^30^. For example, to be sensitive only to nonconservative force, the polarizability of the probe particle should be purely imaginary. This condition can be satisfied if one uses certain absorbing materials with dielectric permittivity 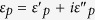 satisfying the following expression (see Supplementary Materials for derivation):


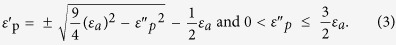


Dielectric permittivity of some metals, such as silver, copper, and boron, in air can satisfy this condition in ultraviolet (see Supplementary Materials). For metallic (gold) nanoparticles in water this condition is satisfied in the near-infrared region (*λ* = 980 nm)^31^. Another possibility is to use gold particles in a liquid with high refractive index (e.g. bromonaphtalene) at *λ* = 530 nm^32^. For silver nanoparticles in water, condition (3) can be achieved in ultra-violet region (*λ* = 394 nm)^32^. For other spectral ranges, condition (3) can be satisfied using combination of materials. Thus, by choosing the material of the particle in accordance with Eq. (3), one can make the particle to be sensitive only to nonconservative forces.

It can be shown using vector identities and Maxwell’s equations that equation for optical forces (1) is equivalent to the following one^26^,^33^


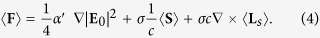


Here 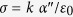 is the total cross section of a dipolar particle, 
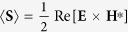
 is the time averaged Poynting vector, 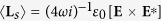 is the time averaged spin density of a transverse electromagnetic field. This expression for the force 〈**F**〉 is more convenient than Eq. (1) as it explicitly divides optical force into conservative (the first term in the right hand side of Eq. (4)) and nonconservative (the second and the third terms) parts. It has been previously shown^16^ that the field perturbation due to the probe particle does not drastically change the surrounding electromagnetic field inside the similar structure of periodic rods. This conclusion remains valid even for highly scattering metallic particles^16^. Thus, we will use Eq. (1) in the following to calculate optical forces on a dipole particle inside photonic crystals.

## Probe particle within a left-handed photonic crystal

As one can see from Table 1, negative refractive index in 2D photonic crystals can be achieved for both TE- (electric field of incident wave is perpendicular to the rods) and TM- (electric field is parallel to the rods) polarizations.

Due to symmetry of the problem, in TM case, electric field inside the PhC has the only component parallel to the rods. Thus, **E** × E^*^ ≡ 0 and the last term in Eq. (4) becomes zero. In the absence of the first term, the optical force becomes proportional to the Poynting vector and thus the force is always positive. Similar result was found in a case of plasmonic photonic crystals^31^. Even though probe particles in that work experienced “negative refraction”, they were moving along the energy flow.

In the case of TE-polarized waves, the third term in Eq. (4) is generally nonzero. We investigate the phase velocities and the corresponding forces in several photonic crystals that were previously identified as left-handed and negatively refracting. The force determination requires careful calculation of electromagnetic fields inside PhC. These calculations were performed using finite-elements method (COMSOL Multiphysics) using exactly the same parameters as in original papers (see Table 1). For this paper, we consider two cases: a PhC with square and triangular lattices irradiated by a TE wave.

## Square lattice PhCs

**Figure 2:**
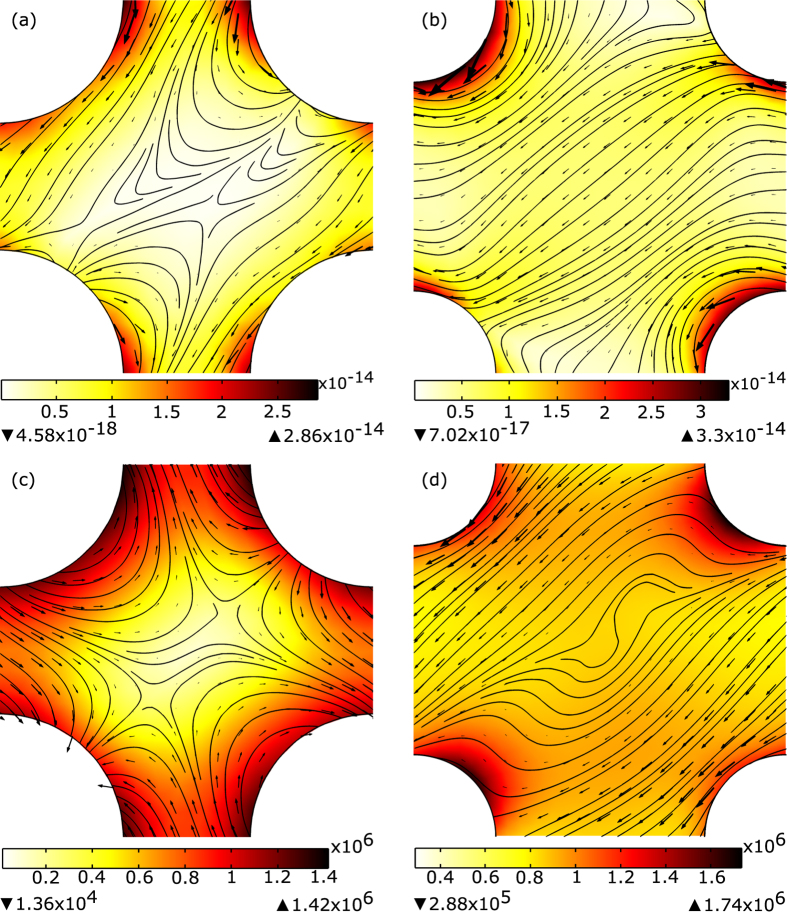
One unit cell of the left-handed PhC with square lattice and parameters taken from ref. 20 (**a** and **c**), and ref. 21 (**b** and **d**) - see Table 1. Figures (**c** and **d**) show the plot of the electric field amplitude (color map) and power flow (arrows), while Figures (**a** and **b**) show the optical force magnitude (color map), direction (arrows), and possible trajectories (lines) for the probe particle. The interior of the rods is not shown.

The first structure is based on the PhC in the paper of Gajic, *et al*.^20^. PhC consists of a square array of rods with permittivity ε_r_ = 9.61, fill ratio *r*/*d* = 0.33 with *r* being the radius and *d* being the lattice constant (Fig. 1). External TE polarized wave with normalized frequency 
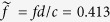
 (*c* is the speed of light) is incident at an angle of 15°. We were able to recreate the simulations of ref. 20 such that the phase velocity of the wave inside the PhC is negative, and the wave is propagating towards the interface (see Supplementary Media). The optical force was calculated for a spherical particle with radius (*c*/*f*)/10 and dielectric permittivity ε_*p*_ = 0.679 + 0.825*i*, which corresponds to the properties of silver at wavelength *λ*_0_ = 0.317 *μm*. The small dimensions of the probe particle allow to use dipolar approximation and the chosen dielectric permittivity ensures sensitivity of the probe only to nonconservative optical forces. We show the power flow and forces calculated with Eq. (1) in Fig. 2a and c. The power flow has complex pattern moving in the loops through and around the rods, but overall propagation is down and to the left (negative refraction). Comparing Fig. 2a and c, one can see that the force field does not follow the flow of enegry. This is the consequence of nonzero spin density term in Eq. (4). As one can see in Fig. 2a, the direction of the optical force is down (away from the interface) and to the left that corresponds to the negative refraction, but not to the negative force.

We simulated a second square lattice PhC with left-handed properties originally proposed by Derbali and AbdelMalek^21^, with relative permittivity *ε*_*r*_/*ε*_*a*_ = 8.9, normalized frequency 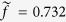, fill ratio *r*/*d* = 0.22, and angle of incidence for TE polarized wave is 10°. The resulting power flow and forces are shown in Fig. 2b and 2d. Similar to the previous structure, here we see that the horizontal component of the force is directed opposite to the incident field (negative refraction), but vertical component of the force is directed away from the surface (not negative).

## Triangular lattice PhCs

**Figure 3:**
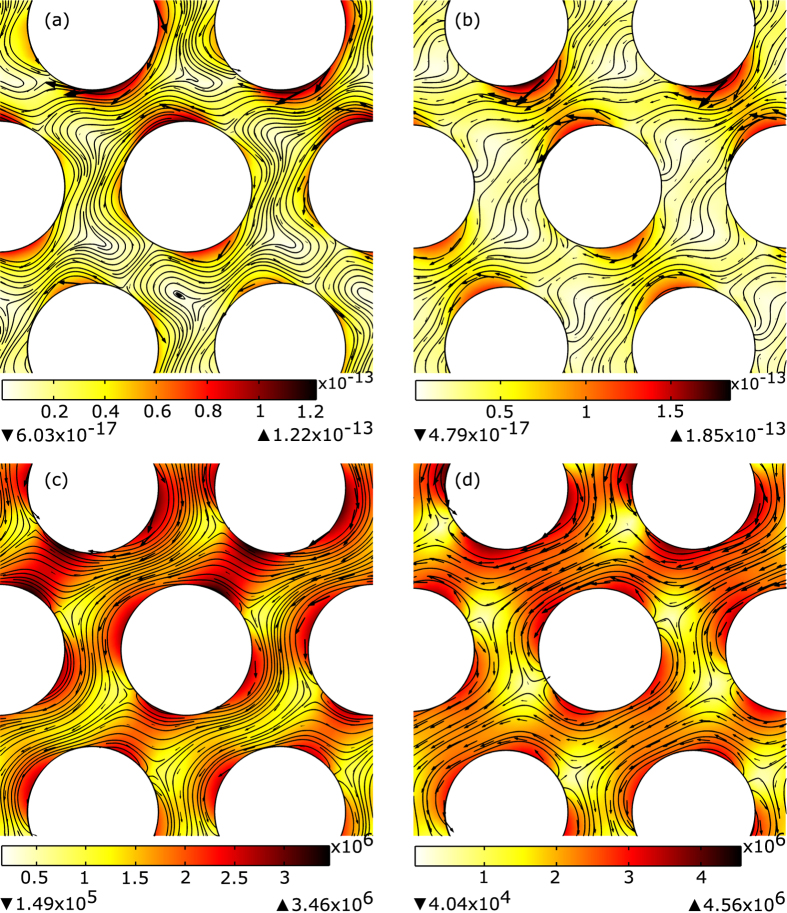
Unit cell of PhC with a triangular lattice with parameters as in ref. 10 (**a** and **c**), and ref. 24 (**b** and **d**) - see Table 1. Figures (**c** and **d**) show the plot of the electric field amplitude (color map) and power flow (arrow surface), while Figures (**a** and **b**) show the optical force magnitude (color map), direction (arrow surface), and possible trajectories (lines) for the realistic silver probe. Similar to Fig. 2, the horizontal component of the force is opposite to the incident field inside the crystal.

The second structure we considered was a triangular lattice, also with an incident TE wave. The first case is based on the paper of Foteinopoulou *et al*.^10^; a triangular array of rods with permittivity 12.96 and *r*/*d* = 0.35. The field with TE polarization and with normalized frequency 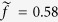 is incident at an angle of 30°. The power flow and optical forces are shown in Fig. 3a and c. Figure 3a again demonstrates “negative refracted forces”, but no negative forces.

Another triangular lattice is based on the paper of Guven *et al*.^24^, using rods with dielectric constant 9.61 in air and fill ratio of 0.329. In their experiment, they used normalized frequency of 0.667, and TE-polarized wave was incident at an angle of 30°. The results are shown in Fig. 3b and d. Similar to the previous figures, the horizontal component of the force outside the crystal is also opposite to the force inside the crystal, but negative forces are absent.

## Discussion and Conclusions

**Figure 4:**
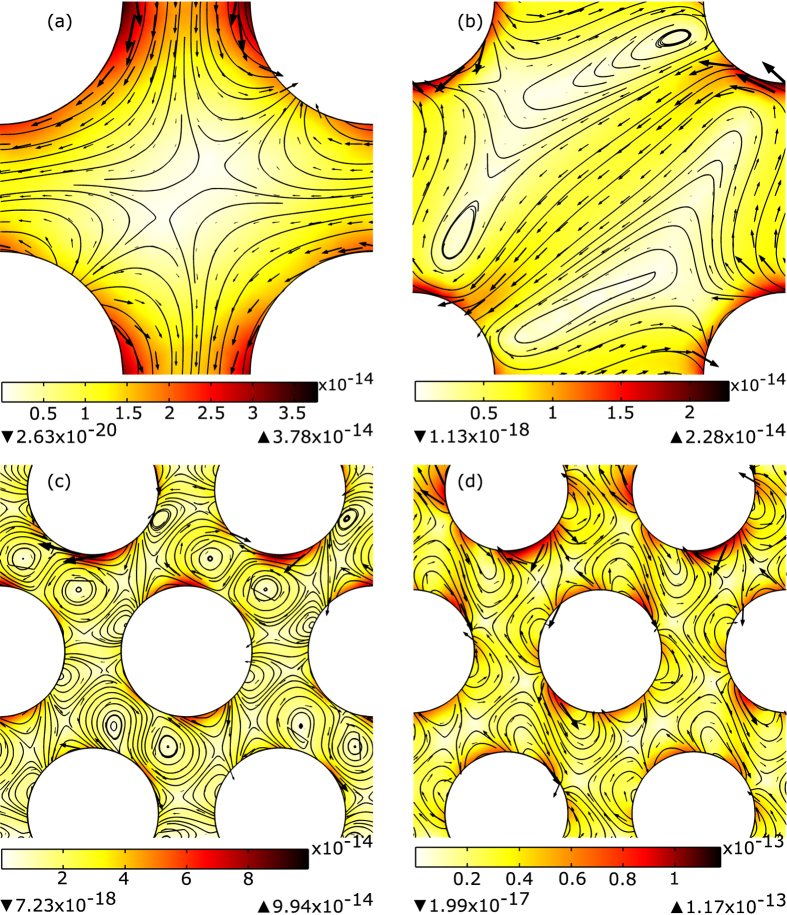
Curl spin force (the third term of Eq. (4) for the left-handed PhC considered in present paper. (**a**) ref. 20; (**b**) ref. 21; (**c**) ref. 10; (**d**) ref. 24.

The analysis of the optical forces inside left-handed PhC performed in the previous section shows that the force may not follow the direction of power flow, but nevertheless it remains positive. Still the question remains if one can find parameters for PhC to revert the force. To clarify this question, we should notice that the negative force can appear only because of action of the third term in Eq. (4). If this force is negative, but smaller than the part of the force corresponding to the energy flow (the second term in Eq. (4)), there is a hope to increase this term by corresponding choice of PhC parameters to achieve negative total force. In Fig. 4, curl spin forces are shown for the cases of PhC considered in previous section. One can see that, in general, curl spin force does not help in achieving negative forces. In the cases corresponding to refs 20 and 21, the force is directed away from the surface; in the cases of refs 10 and 24 one can observe circulating behavior. Overall, in all considered examples there are no cases when curl spin force is negative (directed towards the surface) which, to our opinion, makes the possibility of achieving negative forces for dipole size particles in left-handed PhC highly improbable.

One of the reasons for different behavior of forces inside homogeneous LHM and left-handed PhCs is that force on a probe particle is determined not only by electromagnetic field momentum, but also by the momentum transfer from a surrounding medium^4^. This reaction of the medium is present in LHM, but is absent when the probe particle is in air.

The absence of negative forces for dipole size particles does not mean that these forces are absent in general case of large particles. The PhC environment provides unique possibilities for modification of scattering from finite size particles. Thus, favorable forward scattering conditions for implementing ‘tractor beams’ can be achieved in analogy with the free space approaches^34^,^35^,^36^. However, this problem requires separate extended research.

In conclusion, we calculated nonconservative optical forces acting on a dipole particle inside two-dimensional left-handed photonic crystals proposed in currently available literature. In left-handed metamaterials, the existence of negative forces (forces opposite to the energy flow) was predicted previously^1^,^4^,^5^. However, we proved that negative force cannot exist for 2D PhC illuminated with TM polarized incident field. When insensitive to the gradient forces, the probe particles inside these structures follow the flow of energy. Extensive numerical calculations for the PhC structures illuminated with TE polarized light also do not show the presence of negative forces. Although in this case the energy flow and the force field are not collinear, the force field is still directed away from the interface of PhC. In this way we addressed the very important question about electromagnetic momentum and optical forces inside exotic (left-handed in current case) materials. Our paper shows that even though photonic crystals can imitate the properties of isotropic left-handed materials (negative refraction, negative phase propagation, flat-lens focusing) in some respect, they are not their full equivalent and extrapolation of physical properties between them should be made with caution.

## Methods

### The details of numerical simulations

Calculations of electromagnetic fields inside two-dimensional photonic crystals were performed using finite-elements method (COMSOL Multiphysics 5.1). The unit cell of the 2D-PhC structure with corresponding lattice was created, using parameters of Table 1. To reproduce semi-infinite photonic crystal, the unit cell was replicated 10 times in vertical direction; to prevent the wave reflection from the bottom of PhC, a small dielectric loss (Im*ε* = 0.05) was added to the permittivity to the rods. Periodic boundary conditions were set on the left and right hand sides of the simulation domain, for the system to appear infinite. At the top and bottom edges, periodic ports were created, with the top edge having the plane wave excitation. The mesh was set up such that the left and right edge meshing was identical, whereas everywhere else, a free triangular mesh with minimum element size 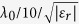 was used. Finally, the variables for the forces were set in COMSOL according to Eq. (1). Separate conservative and nonconservative force components were calculated according to Eq. (4).

## Additional Information

**How to cite this article:** Ang, A. *et al*. Scattering Forces within a Left-Handed Photonic Crystal. *Sci. Rep.* **7**, 41014; doi: 10.1038/srep41014 (2017).

**Publisher's note:** Springer Nature remains neutral with regard to jurisdictional claims in published maps and institutional affiliations.

## Supplementary Material

Supplementary Materials

Supplementary Video

## Figures and Tables

**Table 1:** A list of the photonic crystals demonstrating negative refractive index.

Reference	Polarization	Lattice type	Fill ratio, *r*/*d*	Permittivity of the rods *ε*_*r*_	Incident Angle, *θ*	Normalized frequency, 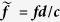
20, (Fig. 6 therein)	TE	square	0.33	9.61	15°	0.413
21	TE	square	0.22	8.9	10°	0.732
10	TE	triangular	0.35	12.96	30°	0.58
24	TE	triangular	0.329	9.61	30°	0.667
23	TM	square	0.329	9.61	30,45,60	0.2188
22	TM	square	0.175	9.2	n/a	0.72

For all cases, the permittivity of the host material *ε*_*a*_ is unity.
