# Supplementary material for: Scattering Forces within a Left-Handed Photonic Crystal
Source: Sci Rep. 2017 Jan 23;7:41014. doi: 10.1038/srep41014 (PMC5253622; doi:10.1038/srep41014)
Supplement: Supplementary Materials [file srep41014-s1.pdf]

Supplementary Materials

Scattering Forces within a Left-Handed Photonic Crystal

A. Ang*, S. Sukhov, A. Dogariu, A. Shalin

I. Optical force inside homogeneous material with negative refractive index

Forces on a small particle inside homogeneous metamaterial with negative refractive index can be calculated explicitly using the expression for the force on a dipole (Eq. (1) of the main text). The results of such calculations are shown in Figure S1. These results demonstrate that optical forces inside left-handed material are negative and directed towards the interface against the power flow.

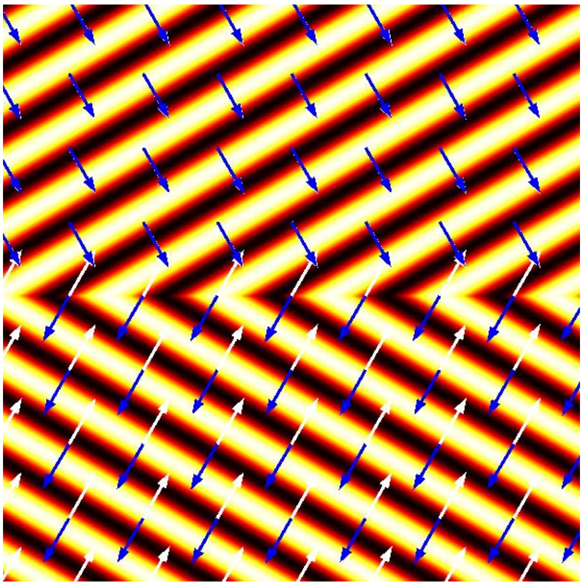

Figure S1: Power flow and forces in a homogeneous left-handed material. For the lower half of this figure, $\epsilon = \mu = -1$. The white arrows indicate the optical forces, whereas blue ones indicate the power flow. The phase velocity of the wave and optical forces in the lower half of the material is directed opposite to the power flow.

II. Dipole probe particle with pure imaginary polarizability

According to Clausius-Mossotti relation, the probe particle of radius *r* and relative permittivity ϵ_p has polarizability[1,2]

$$\alpha = 4\pi r^3 \cdot \epsilon_0 \frac{\epsilon_p - \epsilon_a}{\epsilon_p + 2\epsilon_a}, \qquad \text{(S1)}$$

where ϵ_a is the relative permittivity of the surrounding environment, which we assume to be purely real-valued. We write ϵ_p as a complex number $\epsilon_p' + i\epsilon_p''$. Strictly speaking, Eq.(S1) should

* Corresponding author: angeleene.ang@gmail.com

also contain radiative correction $\sim k^3\alpha$ [1], however, in a case of complex dielectric permittivities ε_p this correction is small and can be omitted. With this in mind, we can separate α into real and imaginary parts

$$\alpha = 4\pi r^3 \cdot \epsilon_0 \left[\frac{(\epsilon_p')^2 + (\epsilon_p'')^2 - 2\epsilon_a^2 + \epsilon_a \epsilon_p'}{\left(\epsilon_p' + 2\epsilon_a\right)^2 + \left(\epsilon_\mathrm{p}''\right)^2} + i \frac{3\epsilon_a \epsilon_p''}{\left(\epsilon_p' + 2\epsilon_a\right)^2 + \left(\epsilon_\mathrm{p}''\right)^2} \right]. \tag{S2}$$

The probe particle would sense only nonconservative optical force if the real part of α is zero, and the imaginary part to be non-zero (see the main text). Hence, we can conclude that for our case,

$$\epsilon_a \epsilon_p'' \neq 0 \tag{S3}$$

To restrict further, we also need to find values of ϵ_p such that

$$\mathrm{Re}(\alpha) = 4\pi r^3 \cdot \epsilon_0 \frac{(\epsilon_p')^2 + (\epsilon_p'')^2 - 2\epsilon_a^2 + \epsilon_a \epsilon_p'}{\left(\epsilon_p' + 2\epsilon_a\right)^2 + \left(\epsilon_\mathrm{p}''\right)^2} = 0. \tag{S4}$$

Equating the numerator of Eq. (S4) to zero, one can get the following expression for the real part of the particle permittivity:

$$\epsilon_p' = \pm\sqrt{\frac{9}{4}\epsilon_a^2 - (\epsilon_p'')^2} - \frac{1}{2}\epsilon_a . \tag{S5}$$

Finally, the permittivity needed to obtain a purely imaginary polarizability needs to be of the following form

$$\epsilon_\mathrm{p} = \pm\sqrt{\frac{9}{4}\epsilon_a^2 - (\epsilon_p'')^2} - \frac{1}{2}\epsilon_a + i\epsilon_p'' \text{ , where } 0 < \epsilon_p'' \leq \frac{3}{2}\epsilon_a . \tag{S6}$$

The condition (S6) is satisfied by Boron[3], Copper[4], and Silver[5] in the ultraviolet spectral range, if the environment were air or vacuum ($\varepsilon_a = 1$) (Figure S2). In the main text, we used silver as the probe particle's material, with permittivity of $\epsilon_p = 0.679 + 0.825i$ in the frequency $f_0 = 945\,\mathrm{THz}$ (0.317μm wavelength).

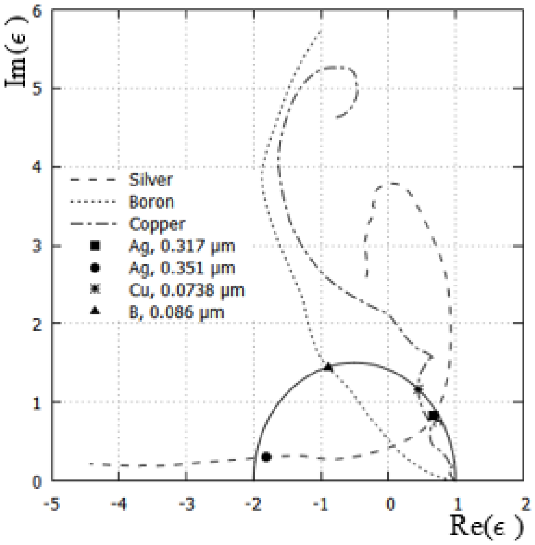

Figure S2: Plot of the real part of dielectric permittivity $\mathrm{Re}\,\varepsilon$ versus its imaginary part $\mathrm{Im}\,\varepsilon$ with a locus of points (solid black line) specifying zero real part of polarizability of the probe particle (Eq. (S6)). The points indicate the permittivities for zero real polarizability: for silver, the point (0.679,0.825) corresponds to λ = 0.317 μm wavelength and the point (-1.814,0.297) corresponds to λ = 0.351 μm; for copper, the point (0.447,1.16) corresponds to λ = 0.074 μm; and for boron, the point (-0.888,1.439) corresponds to λ = 0.086 μm.

III. Media file description

Video shows the behavior of magnetic field inside a 2D photonic crystal with square lattice structure (white circles) with parameters taken from Ref.[20] of the main text. TE polarized plane wave is incident from the top of the structure. Red (blue) areas correspond to the regions where magnetic field vector is directed out of (inward to) the screen. Clear phase propagation towards the surface can be observed.

References

1. Draine, B. T. The Discrete-Dipole Approximation and its Application to Interstellar Graphite Grains. *Astrophys. J.* **333,** 848–872 (1988).
2. Chaumet, P. C. & Nieto-Vesperinas, M. Electromagnetic force on a metallic particle in the presence of a dielectric surface. *Phys. Rev. B* **62,** 11185 (2000).
3. Fernández-Perea, M. *et al.* Optical constants of electron-beam evaporated boron films in the 6.8-900 eV photon energy range. *JOSA A* **24,** 3800–3807 (2007).

4. Hagemann, H.-J., Gudat, W. & Kunz, C. Optical constants from the far infrared to the x-ray region: Mg, Al, Cu, Ag, Au, Bi, C, and Al2O3. *J. Opt. Soc. Am.* **65,** 742 (1975).
5. Johnson, P. B. & Christy, R.-W. Optical constants of the noble metals. *Phys. Rev. B* **6,** 4370 (1972).
